# Soil bacterial diversity patterns and drivers along an elevational gradient on Shennongjia Mountain, China

**DOI:** 10.1111/1751-7915.12288

**Published:** 2015-05-29

**Authors:** Yuguang Zhang, Jing Cong, Hui Lu, Guangliang Li, Yadong Xue, Ye Deng, Hui Li, Jizhong Zhou, Diqiang Li

**Affiliations:** 1 Institute of Forestry Ecology, Environment and Protection, Key Laboratory of Forest Ecology and Environment of State Forestry Administration, The Chinese Academy of ForestryBeijing, 100091, China; 2 Research Center for Eco-Environmental Science, Chinese Academy of SciencesBeijing, 100085, China; 3 Institute for Environmental Genomics, Department of Botany and Microbiology, The University of OklahomaNorman, OK, 73019, USA; 4 State Key Laboratory of Forest and Soil Ecology, Institute of Applied Ecology, Chinese Academy of SciencesShenyang, 110016, China; 5 School of Mineral Processing and Bioengineering, Central South UniversityChangsha, 410083, China

## Abstract

Understanding biological diversity elevational pattern and the driver factors are indispensable to develop the ecological theories. Elevational gradient may minimize the impact of environmental factors and is the ideal places to study soil microbial elevational patterns. In this study, we selected four typical vegetation types from 1000 to 2800 m above the sea level on the northern slope of Shennongjia Mountain in central China, and analysed the soil bacterial community composition, elevational patterns and the relationship between soil bacterial diversity and environmental factors by using the 16S rRNA Illumina sequencing and multivariate statistical analysis. The results revealed that the dominant bacterial phyla were *Acidobacteria*, *Actinobacteria*, *Alphaproteobacteria*, *Betaproteobacteria*, *Gammaproteobacteria* and *Verrucomicrobia*, which accounted for over 75% of the bacterial sequences obtained from tested samples, and the soil bacterial operational taxonomic unit (OTU) richness was a significant monotonous decreasing (*P* < 0.01) trend with the elevational increasing. The similarity of soil bacterial population composition decreased significantly (*P* < 0.01) with elevational distance increased as measured by the Jaccard and Bray–Curtis index. Canonical correspondence analysis and Mantel test analysis indicated that plant diversity and soil pH were significantly correlated (*P* < 0.01) with the soil bacterial community. Therefore, the soil bacterial diversity on Shennongjia Mountain had a significant and different elevational pattern, and plant diversity and soil pH may be the key factors in shaping the soil bacterial spatial pattern.

## Introduction

Understanding patterns of biological diversity along elevational gradients and the factors driving such patterns are indispensable to gaining a comprehensive understanding of the response of ecosystems to global climate change, aid in determining the broad scale distributions of species and in the development ecological theories (Lomolino, 2001; Rahbek, 2005; Malhi *et al*., 2010; Wang *et al*., 2011). In the past, most studies have focused on plants and animals (Lomolino, 2001; McCain, 2005; Renaud *et al*., 2009), although soil microbes are abundant and play important roles in ecosystems as decomposers, primary producers and drive many important biogeochemical cycles. Because traditional methods fail to allow researchers to adequately classify microbial communities, little information is available related to the patterns of diversity in soil microbes along elevational gradients.

Recently, the development of techniques related to molecular biology which use the extraction of DNA from environmental samples has allowed researchers to classify soil microbes in different environments and promoted an understanding of how soil microbial diversity changes along elevational gradients (Bryant *et al*., 2008; Fierer *et al*., 2011; Wang *et al*., 2012a; Shen *et al*., 2013). These studies and reports yielded different results related to elevational patterns of microbial communities showing either no trend (Fierer *et al*., 2011; Wang *et al*., 2012a; Shen *et al*., 2013), a monotonous decrease (Bryant *et al*., 2008), an increase (Wang *et al*., 2011) or a humpbacked trend (Singh *et al*., 2012a,b) for soil microbial communities. However, some studies suffered from certain basic limitations. For example, some studies assessed only one or several particular phyla (Bryant *et al*., 2008; Singh *et al*., 2012b) while other studies mixed different latitudes, climatic zones or geological substrates (Lauber *et al*., 2009). Therefore, the primary question of how microbial diversity changes along an elevational gradient and whether such a pattern resembles the patterns observed for macroorganisms remains unanswered (Shen *et al*., 2013). To gain a better understanding, additional studies related to the changes in microbial patterns along elevational gradients are needed.

Shennongjia Mountain (31°15′–31°57'N and 109°59′–110°58'E) stands in northwestern Hubei Province as the highest peak (3105.4 m above sea level) in central China (Ma *et al*., 2008). The unique geographical location and complex terrain of this region makes it one of three centres having the highest biodiversity in China. The region includes the most well-preserved subtropical virgin and mature forest in a mid-latitudinal area on earth. The vertical distribution of vegetation on Shennongjia Mountain transitions very distinctly from evergreen broadleaved forest at low elevations to subalpine shrub at high elevations (Zhao *et al*., 2005). Therefore, Shennongjia Mountain presents an ideal location to study elevational patterns of animal, plant and soil microbial diversity. At present, some studies have shown that plant diversity exhibited a single peak distributional pattern with increasing elevation (Shen *et al*., 2004; Zhao *et al*., 2005; Cong *et al*., 2013).

This study addressed three questions: (i) What is the composition and structure of soil bacterial communities on Shennongjia Mountain? (ii) How do bacterial diversity and richness vary along the mountainside elevational gradient? and (iii) What environmental factors drive the elevational patterns observed in the soil bacterial community? To answer these questions, we selected four typical plant community types along the elevation gradient from 1000 to 2800 m, including evergreen broadleaved forest (EBF1050), deciduous broadleaved forest (DBF1750) and coniferous forest (CF2550) as well as a subalpine shrub (SAS2750) (Table 1), and analysed the soil bacterial diversity using a 16S rRNA Illumina sequencing technique.

**Table 1:** Site information and soil biogeochemical characteristic in this study

Study site	EBF1050	DBF1750	CF2550	SAS2750
Elevation	1009–1057 m	1725–1844 m	2530–2590 m	2720–2776 m
Vegetation types	Evergreen broadleaved forest	Deciduous broadleaved forest	Coniferous forest	Subalpine shrub
Soil organic carbon (g kg^−1^)	52.58 ± 10.21b	28.22 ± 1.01a	59.51 ± 5.25b	60.95 ± 4.98b
Total nitrogen (g kg^−1^)	4.14 ± 0.63b	1.83 ± 0.12a	4.22 ± 0.30b	4.50 ± 0.36b
Available nitrogen (g kg^−1^)	0.29 ± 0.03b	0.18 ± 0.01a	0.33 ± 0.03bc	0.42 ± 0.04c
The ratio of SOC to TN	12.25 ± 0.53a	15.74 ± 0.73c	14.00 ± 0.50b	13.52 ± 0.13ab
Soil pH	6.58 ± 0.32c	5.36 ± 0.19b	4.99 ± 0.05b	4.38 ± 0.03a
Soil moisture (%)	37.47 ± 2.30b	49.13 ± 1.69c	44.52 ± 1.60c	28.86 ± 1.10a
Soil temperature at a 10 cm depth (°C)	20.02 ± 1.78d	16.71 ± 0.12c	10.83 ± 0.18a	12.07 ± 0.35b
Shannon index of plant	2.73 ± 0.16c	2.41 ± 0.14bc	2.10 ± 0.09b	0.84 ± 0.10a

Data present the mean value and standard error (*n* = 8). The same lowercase letters within the same row mean the difference was not significant, whereas the difference was significant *P* < 0.05.

## Results

### Overall soil bacterial community composition and structure

A total of 1 122 910 quality 16S rRNA sequences were detected in all study sites, with 21 098–49 741 (mean 35 090) sequences per sample. The complete linkage clustering method was used to define operational taxonomic units (OTUs), using 97% identity as a cut-off and resulting in 79 494 OTUs being detected. Rarefaction analysis and Chao1 estimator showed that the diversity in these soil samples was within the same range (Fig. S1, Table 2).

**Table 2:** Summary of OTU number, Chao 1, Shannon and Simpson indices based on relative abundance of bacterial sequences at four different sites

Sample	No. of OTUs (0.03)	Chao1	Shannon index	Simpson index
EBF1050	8208.38 ± 269.82c	18284.82 ± 1382.19ab	8.14 ± 0.09c	894.37 ± 163.85b
DBF1750	7490.50 ± 154.72b	20801.44 ± 1531.48b	7.75 ± 0.04b	330.32 ± 65.76a
CF2550	6544.00 ± 272.93a	18494.40 ± 1361.45ab	6.99 ± 0.19a	82.96 ± 37.67a
SAS2750	6250.50 ± 187.08a	15972.55 ± 547.60a	7.43 ± 0.09b	253.66 ± 70.68a

Data present the mean value and standard error (*n* = 8). The same lowercase letters within the same row mean the difference was not significant, whereas the difference was significant *P* < 0.05.

The classified phylotypes were counted at different taxonomical levels and 36 bacterial phyla were presented in these forest sites (Table S1), and all phylotypes were detected at all sample sites. Ninety class and 153 order were found in the tested samples (Table S1). The relatively abundant dominant phyla were *Acidobacteria*, *Actinobacteria*, *Alphaproteobacteria*, *Betaproteobacteria*, *Gammaproteobacteria* and *Verrucomicrobia*, which accounted for more than 75% of the bacterial sequences from each of the soils (Table S2). In addition, *Bacteroidetes*, *Planctomycetes*, *Firmicutes* and *Deltaprotecobacteria* were presented in most soils but at low relatively abundance, and the other phyla were rarely found in these soil samples (Table S2). The OTUs number at different taxonomical level were counted and showed the different dominant phylotypes were existed (Tables S3 and S4). For example, the *Alphaproteobacteria* is the most dominant phylotype at the class level, and the *Rhizobiales* is the most dominant phylotype at the order level (Table S4).

**Fig 1:**
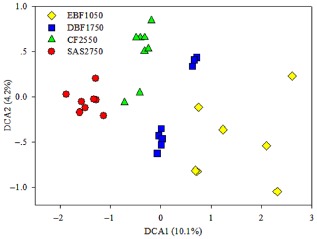
Detrended correspondence analysis of soil bacteria community structure based on the relative abundance of detected bacterial sequence number.

Detrended correspondence analysis showed that four distinct clusters were formed and were well separated from among samples (Fig. 1). The results of the Multi-Response Permutation Procedure, Adonis and Anosim also showed significant differences (*P* < 0.01) existed among different study sites (Table S5). Therefore, the soil bacterial communities were sharply different among these study sites.

### Bacterial community distribution pattern along an elevational gradient

**Fig 2:**
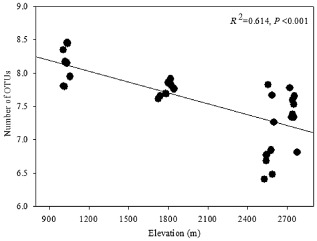
The regression relationship between soil microbial OTUs richness and elevation.

The soil bacterial community was analysed by calculating the Shannon and Simpson indices, and OTUs numbers (richness). The soil bacterial Shannon index ranged from 6.99 ± 0.19 to 8.14 ± 0.09, Simpson index ranged from 82.96 ± 37.67 to 894.37 ± 163.85, and the number of OTUs ranged from 6250.50 ± 187.08 to 8208.38 ± 269.82 in these four sites respectively (Table 2). The results showed that the soil bacterial Shannon index decreased significantly (*P* < 0.05) with increasing elevation from EBF1050 to CF2550 (Table 2). However, the richness monotonously decreased significantly (*R*^2^ = 0.614, *P* < 0.01) with increasing elevation (Table 2, Fig. 2).

**Fig 3:**
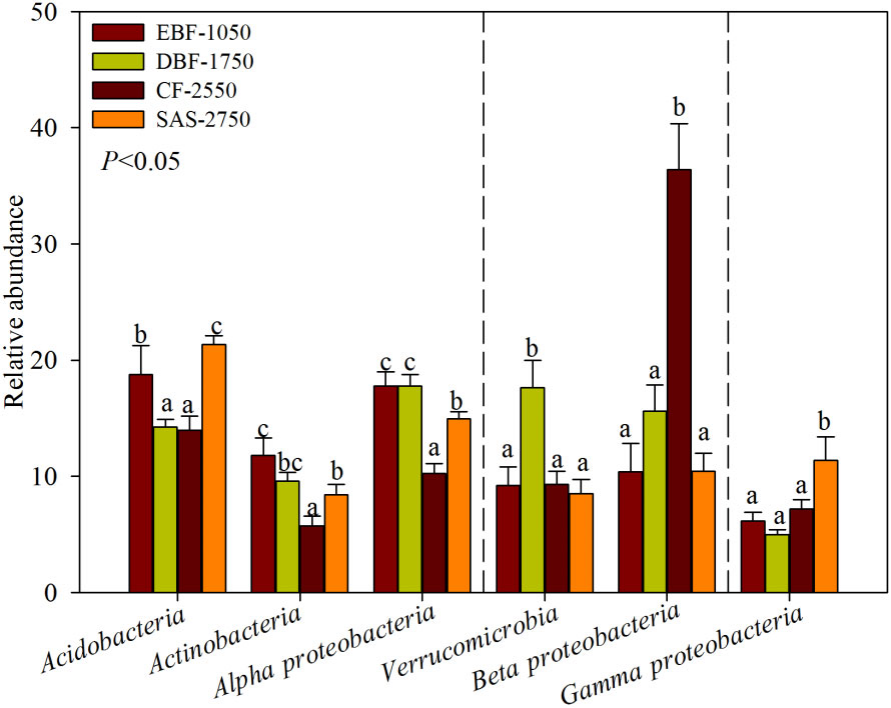
Relative abundance of the dominant bacterial phyla in soil separated according to study sites. All data are presented as mean± SE. Different letters indicated statistical differences at a P value of < 0.05 among sampling sites by ONE ANOVA.

The relative abundance of bacteria at the phylum level varied among the four sites. We documented distributional patterns of relative abundance for the six dominant phyla (Fig. 3). One of three trends were observed for each phylum: (i) the relative abundance decreased from EBF1050 to CF2550 and increased in the SAS2750 sites, such as *Acidobacteria*, *Actinobacteria*, *Alphaproteobacteria*; (ii) the relative abundance increased from EBF1050 to CF2550 and decreased SAS2750, such as *Verrucomicrobia* and *Betaproteobacteria* and (iii) no significant difference was observed, such as *Gammaproteobacteria*. Therefore, the relative abundance of the dominant phyla showed different elevational patterns (Fig. 3). In addition, the richness also exhibited similar trends in different dominant phyla (Table S3). The different phyla were dominant at different study sites (Fig. 3, Table S2). For example, the most dominant phyla were *Acidobacteria* (18.85%) and *Alphaproteobacteria* (17.78%) in EBF1050, *Alphaproteobacteria* (17.77%) and *Verrucomicrobia* (17.63%) in DBF1750, *Betaproteobacteria* (36.41%) in CF2550 and *Acidobacteria* (21.34%) in SAS2750.

### Bacterial elevational distance-decay patterns

**Fig 4:**
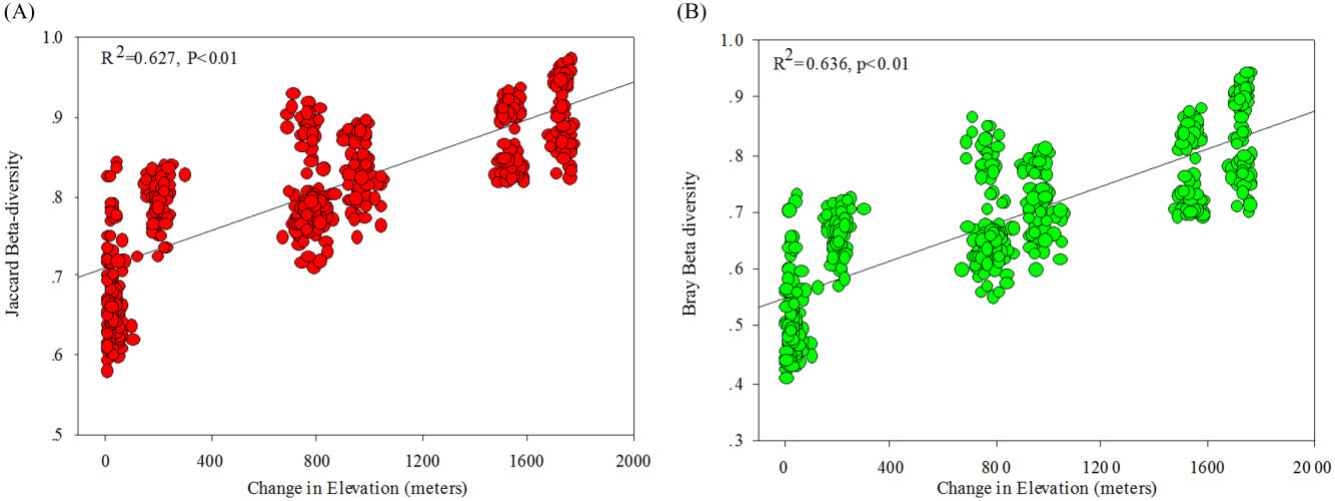
The regression relationship between the beta diversity of Jaccard (A) and Bray–Curtis (B) index and change in elevation distance.

To detect and compare the level of beta diversity among different sites along an elevational gradient, we analysed the beta diversity using Jaccard and Bray–Curtis indices (Fig. 4). The Jaccard and Bray–Curtis indices ranged from 0.52 to 0.63 and 0.42 to 0.50 within the study sites and 0.78 to 0.91 and 0.64 to 0.84 among the sites respectively (Table S6). The pairwise bacterial compositional dissimilarities across the elevational distance increased significantly (*P* < 0.01) with increasing elevation (Fig. 4). Thus, the soil bacterial diversity on Shennongjia Mountain showed a significant elevational distance-decay.

### Linking bacterial community structure and environmental factors

Mantel tests and canonical correspondence analysis (CCA) were performed to analyse the major environmental factors that shaped the bacterial community and structure. The results of a partial Mantel test showed that the bacterial community and structure were significantly linked (*P* < 0.01) to the soil and plant factors (Table 3). For example, the *Acidobacteria*, *Actinobacteria*, *Firmicutes*, *Alphaproteobacteria*, *Betaproteobacteria* and *Planctomycetes* exhibited a significant positive relationship (*P* < 0.05) with soil and plant factors, while *Bacteroidetes*, *Verrucomicrobia* and *Deltaproteobacteria* were significant positively linked (*P* < 0.05) with soil factors. Therefore, the soil bacterial community and structure at the phylum level was controlled by different environmental factors.

**Table 3:** Partial Mantel analysis on the relationship between the relative abundance of dominant phyla and soil characteristics or plant properties

Phylum	Soil,^a^ partial plant		Plant,^b^ partial soil	
	r	*P*	r	*P*
All phylotypes	0.634	0.001	0.330	0.001
*Acidobacteria*	0.578	0.001	0.196	0.023
*Actinobacteria*	0.536	0.001	0.191	0.045
*Bacteroidetes*	0.343	0.002	0.125	0.133
*Chloroflexi*	0.101	0.163	0.444	0.001
*Firmicutes*	0.481	0.001	0.281	0.001
*Alphaproteobacteria*	0.572	0.001	0.319	0.002
*Betaproteobacteria*	0.196	0.015	0.306	0.001
*Deltaproteobacteria*	0.555	0.001	0.105	0.219
*Gammaproteobacteria*	0.389	0.001	0.250	0.004
*Gemmatimonadetes*	0.424	0.001	0.309	0.002
*Planctomycetes*	0.505	0.001	0.159	0.046
*Verrucomicrobia*	0.551	0.001	−0.033	0.635

a Selected soil variables: soil organic carbon, total nitrogen, available nitrogen, soil moisture, soil pH and soil temperature at the depth of 0–10 cm.

b Selected plant variables: the Shannon index of tree and shrub.

**Fig 5:**
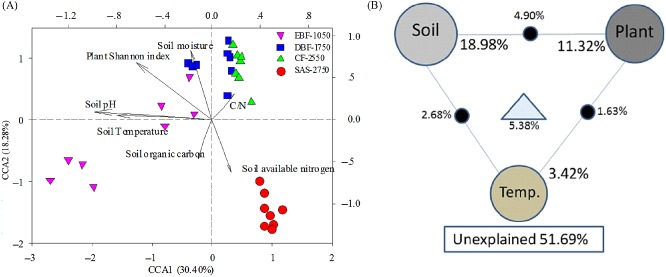
The relationship analysis between soil microbial functional gene diversity and environmental variables.A. Canonical correspondence analysis (CCA) of relative abundance data and soil environmental variables that were significantly related to microbial variations, C/N: the ratio of soil organic carbon to soil total nitrogen.B. Variation partitioning analysis (VPA) of microbial diversity explained by soil chemical factors (soil, including soil organic carbon, available nitrogen, the ratio of soil organic carbon to total nitrogen, soil moisture and pH), soil temperature (temp) and plant diversity (the Shannon index of trees and shrubs), and their relationships. Each diagram represents the biological variation partitioned into the relative effects of each factor or a combination of factors, in which geometric areas were proportional to the percentages of explained variation.

CCA was used to identify the major environmental variables that significantly controlled the soil bacterial community structure (*P* = 0.005). The Shannon index for plants and soil pH appeared to be the most important environmental factors in controlling the bacterial community structure since they had long-term projections, which represented major variations among microbial communities (Fig. 5A). The regression analysis also showed that the number of soil bacterial OTUs was significant linked to plant Shannon index (*R*^2^ = 0.472, *P* < 0.01) and soil pH (*R*^2^ = 0.485, *P* < 0.01) (Figs S2 and S3). Therefore, soil temperature may directly affect the relative abundance of soil microbes, and soil pH and plant diversity may directly control the bacterial species present or absent at any particular site.

Variance partitioning analysis was used to quantify the contributions of soil chemical factors (soil), soil temperature (temp) and plant diversity (plant) to variation in the bacterial community. A total of 48.31% of the variation was explained by these three environmental variables (Fig. 5B). Soil, temp and plant independently explained 18.98%, 3.42% and 11.32% of the variation in the bacterial community structure, respectively, verifying that they were major factors shaping bacterial community structure.

## Discussion

Microorganisms are the most abundant and diverse group of species on our planet and play important roles in the biogeochemical cycling of various materials and elements; however, the spatial pattern of microbial diversity at the community level remains poorly studied (Martiny *et al*., 2011). The development and increasing availability of high-throughput molecular techniques has helped improve our understanding of the composition and spatial distribution of microbial communities at different scales. This is especially true for the use of direct sequencing of metagenomic DNA that has become the most accurate approach for the assessment of the taxonomic composition of microbial communities (Lin *et al*., 2010; Martiny *et al*., 2011). Fierer and Jackson (2006) indicated that *Acidobacteria*, *Actinobacteria*, *Proteobacteria* and *Bacteroidetes* dominated all biomes, and the bacterial community composition does not vary significantly across different biomes. In recently years, more and more reports have shown that the dominant bacterial community compositions in soils are similar in various ecosystems (Fierer *et al*., 2011; Wang *et al*., 2011; Shen *et al*., 2013). For example, *Acidobacteria*, *Alphaproteobacteria*, *Actinobacteria*, *Betaproteobacteria* and *Gammaproteobacteria* accounted for over 75% of the bacteria in Changbai Mountain, in northeastern China (Shen *et al*., 2013). *Acidobacteria* and *Proteobacteria* were dominant in a mountainous forest of eastern Peru (Fierer *et al*., 2011). *Proteobacteria* accounted for more than 50% of the soil bacterial community in pristine perhumid forests in the Yuanyang Lake ecosystem in Taiwan (Myers *et al*., 2001). *Proteobacteria*, *Acidobacteria*, *Actinobacteria* and *Bacteroidetes* are the dominant phyla on Mount Fuji in Japan (Singh *et al*., 2012b). In our study, the *Proteobacteria*, *Acidobacteria*, *Actinobacteria* and *Verrucomicrobia* dominated and accounted for over 75% of all the classified bacterial sequences on Shennongjia Mountain. Simultaneously, the similar forest types may have the same dominant microbial phylum; for example, the *Betaproteobacteria* is the most dominant phylum in the dark-coniferous spruce–fir forest in Changbai Mountain (Shen *et al*., 2013), and in Shennongjia Mountain, the *Acidobacteria* is the most dominant phylum in the broadleaved forest in Peru (Fierer *et al*., 2011) and in Shennongjia Mountain. Therefore, the dominant phyla of soil microbial communities may be similar in various ecosystems; forest types may be one of the key factors shaping the dominant phyla.

Patterns of change in microbial diversity along elevational gradients make an extremely interesting topic among ecologists because of the existence of fundamental biogeographic patterns underlying microbial biogeography (Martiny *et al*., 2011; Wang *et al*., 2012b). Elevation is well known to nearly always be a complex and direct driving factor for different climatic gradients, and climatic factors have been shown to have the strongest positive associations with patterns of elevational diversity for animals and plants (Storch *et al*., 2012b; Forister *et al*., 2010). Elevational patterns on mountainsides may minimize the impact of environmental factors (Singh *et al*., 2012a). At present, studies have produced different results related to elevation patterns of microbial communities; this indicates some other factors may be driving the elevational distribution of microbial communities (Bryant *et al*., 2008; Fierer *et al*., 2011). In our study, four typical forest types were formed along an elevational gradient, and plant diversity had a monotonic decrease with elevation. Both the CCA and relationship analysis showed that plant diversity and soil microbial diversity are significantly (*P* < 0.01) correlated. Therefore, plant diversity may be one of the key impact factors that shapes the microbial community and its diversity along elevational gradients. Plant communities can influence the associated soil microbial communities through the types of communities present, the amounts of carbon and nutrient inputs, and by influencing the temperature and water content of the soil (Myers *et al*., 2001; Waldrop and Firestone, 2004). Different plant species can be associated with different communities as evidenced by fatty acid (Lin *et al*., 2010), physiological (Myers *et al*., 2001) and DNA techniques (Kuske *et al*., 2002). Therefore, the different plant community types that have formed along elevational gradients serve as an important factor in shaping the elevational patterns of the microbial community.

Soil pH is usually one of the best predictors of variation in microbial diversity in the various horizons of soils (Shen *et al*., 2013), including at continental scales (Fierer and Jackson, 2006; Chu *et al*., 2010), in the presence of land use changes (Jenkins *et al*., 2009) and at small scales (Baker *et al*., 2009). Some specific phyla exhibit unique patterns of response to variations in pH. For example, the relative abundance of *Acidobacteria* increased with decreasing pH (Jones *et al*., 2009), while the relative abundance of *Alphaproteobacteria* decreased with increasing pH (Chu *et al*., 2010). Recent reports also indicated that pH has a strong correlation with microbial diversity and composition along an elevational gradient in soils or in other environments. For example, Shen *et al*. (2013) observed soil pH is the primary factor in controlling bacteria diversity and community composition across an elevational gradient on Changbai Mountain (Singh *et al*., 2012b). Xiong and colleagues (2012) revealed pH is a better predictor of bacterial community structure in alkaline sediment than in soils with other pH levels. In our study, CCA and the Mantel test all showed that pH (*P* < 0.01) is an important factor that influenced the elevational patterns at the community level of some microbial phyla. The strong correlation between soil pH and microbial diversity could be a result of soil pH interacting with a number of other individual soil and site variables (Fierer and Jackson, 2006). Some authors have expressed the idea that pH maybe an independent driver of soil microbial diversity because the microorganisms themselves have specific pH values and a significant deviation in pH causes environmental stresses (Madigan *et al*., 1997). Therefore, pH is a universally good predictor of bacterial distribution patterns (Singh *et al*., 2012b).

Beta diversity is central to many ecological and evolutionary topics and is a universal biogeographic pattern observed in communities from all domains of life (Green *et al*., 2004); however, patterns of beta diversity for microorganisms have been left understudied (Wang *et al*., 2012b). At present, several studies have examined the beta diversity of bacterial communities on mountainsides (Bryant *et al*., 2008) and in aquatic environments (Wang *et al*., 2012b). Bryant and colleagues (2008) showed that bacterial lineages of *Acidobacteria* were not randomly distributed but exhibited significant (*P* < 0.05) spatial structure across elevational gradients, and suggested that bacterial lineages harbour increasingly disparate ecological features (or functions) at increased elevational distances as a probable consequence of abiotic filtering. Wang and colleagues (2012b) examined the aquatic microbial beta diversity in Laojun Mountain, China, and indicated that species turnover was mostly related to environmental heterogeneity and spatial gradients including horizontal distance and elevation. In this study, we first observed that bacterial compositional similarity at the whole community level significantly decreased (*P* < 0.01) with elevational distance based on the Jaccard and Bray–Curtis indices. Therefore, these results indicated that bacteria may have a strong ability to disperse to a wide variety of environmental conditions (Wang *et al*., 2012b).

## Conclusions

The soil bacterial diversity and richness patterns along an elevational gradient on Shennongjia Mountain were analysed using the 16S rRNA Illumina sequencing. The dominant phyla were *Acidobacteria*, *Actinobacteria*, *Alphaproteobacteria*, *Betaproteobacteria*, *Gammaproteobacteria* and *Verrucomicrobia*, which accounted for more than 75% of the bacterial sequences. Bacterial diversity decreased significantly (*P* < 0.01) with increasing elevation from 1050 to 2550 m and increased at 2750 m; however, OTUs richness tended to decrease monotonously with increasing elevation. We also found that the soil bacterial compositional similarity decreased significantly (*P* < 0.01) with elevational distance increased based on the Jaccard and Bray–Curtis indices. Partial Mantel tests and CCA analysis showed soil pH and plant diversity may be the key factors in shaping soil bacterial community structure and diversity on Shennongjia Mountain.

## Supporting Information

**Fig. S1.** Rarefaction curves for OTUs were calculated with sequences normalized to 20 000 for each sample using 0.03 distance OTUs. **Fig. S2.** The regression relationship between soil microbial OTUs richness and plant diversity. **Fig. S3.** The regression relationship between soil microbial OTUs richness and soil pH. **Table S1.** The classified phylotypes detected at different taxonomical levels. **Table S2.** Relative abundances of detected phylum in four forest sites at different elevation. **Table S3.** Numbers of detected OTUs at phylum level in four forest sites. **Table S4.** The OTU number of the top 10 dominant phylotypes detected at different taxonomical levels. **Table S5.** Statistical analysis of differences in the microbial community composition and structure between different sites. **Table S6.** Microbial beta diversity of Jaccard and Bray–Curtis index along elevational distance on Shennongjia Mountain. **Appendix S1.** Materials and methods.
