# Supplementary material for: Soil bacterial diversity patterns and drivers along an elevational gradient on Shennongjia Mountain, China
Source: Microb Biotechnol. 2015 May 29;8(4):739–46. doi: 10.1111/1751-7915.12288 (PMC4476828; doi:10.1111/1751-7915.12288)
Supplement: Supplementary file 1 — Fig. S1. Rarefaction curves for OTUs were calculated with sequences normalized to 20 000 for each sample using 0.03 distance OTUs. Fig. S2. The regression relationship between soil microbial OTUs richness and plant diversity. Fig. S3. The regression relationship between soil microbial OTUs richness and soil pH. Table S1. The classified phylotypes detected at different taxonomical levels. Table S2. Relative abundances of detected phylum in four forest sites at different elevation. Table S3. Numbers of detected OTUs at phylum level in four forest sites. Table S4. The OTU number of the top 10 dominant phylotypes detected at different taxonomical levels. Table S5. Statistical analysis of differences in the microbial community composition and structure between different sites. Table S6. Microbial beta diversity of Jaccard and Bray–Curtis index along elevational distance on Shennongjia Mountain. Appendix S1. Materials and methods. [file mbt20008-0739-sd1.docx]

Soil bacterial diversity patterns and drivers along an elevational gradient on Shennongjia Mountain, China

Yuguang Zhang^1*^, Jing Cong ^1^, Hui Lu^1^, Ye Deng[^2^](mailto:2)^，3^, Hui Li^4^, Jizhong Zhou^3^, Diqiang Li [^1^](mailto:lidq@caf.ac.cn)1*)

^1^Institute of Forestry Ecology, Environment and Protection, and the Key Laboratory of Forest Ecology and Environment of State Forestry Administration, the Chinese Academy of Forestry, Beijing 100091, China

^2^Research Center for Eco-Environmental Science, Chinese Academy of Sciences, Beijing 100085, China

^3^Institute for Environmental Genomics and Department of Botany and Microbiology, the University of Oklahoma, Norman OK 73019

^4^State Key Laboratory of Forest and Soil Ecology, Institute of Applied Ecology, Chinese Academy of Sciences, Shenyang 110016, China

**Materials and methods**

*Site and sampling*

The study sites, located in Shennongjia Mountain, had a mean annual air temperature of 7.2 °C and annual precipitation of about 1,500 mm, most of which falls during summer (Ma et al., 2008). The unique vertical distribution of vegetation on Shennongjia Mountain transforms from evergreen broadleaved forest elevations below 1,300 m, deciduous broadleaved forest between 1,500 and 2,200 m, coniferous forest between 2,200 and 2,600 m, and sub-alpine shrubs above 2,600 m; the plant communities here are generally undisturbed by man (Zhao et al., 2005).

In this study, the plant survey and soil collected were permitted by the administrative bureau of Shennongjia National Nature Reserve. Table 1 provides detailed information on the study sites. The dominant plant communities are *Cyclobalanopsis oxyodon* (Miq.) Oerst*, Cyclobalanopsis myrsinaefolia* (Blume) in EBF1050, *Carpinus viminea*, *Quercus aliena var. acuteserrata*, *Fagus engleriana* in DBF1750, *Abies fargesii* Franch in CF2550 and *Rhododendron oreodoxa* in SAS2750. Samples of the mountain yellow brown soil were collected in September, 2011. At each site, eight 20 × 20 m plots were established with about 20 meters between adjacent plots. In each plot, fifteen 0 - 10 cm deep soil cores were collected and composited to obtain about 400 g soil in total; these were mixed thoroughly and plant roots and stones were removed. Soil samples were preserved at - 80 °C until being thawed for DNA extraction.

*Plant diversity and soil geochemical analyses*

Plant diversity was surveyed in each study plot, including the plant species, number of individuals, canopy dimensions of each tree or shrub, and diameter at breast height (1.3 m) of trees (DBH > 5 cm) and shrubs (DBH > 1 cm). Average soil temperature at each plot was measured by placing a long-thermometer (Spectrum, Aurora, IL, USA) probe at 10 cm depth in relatively open patches. Soil moisture, soil pH, total soil organic carbon and nitrogen concentrations and available nitrogen were measured using the same sieved soil core mixtures that were used for DNA extraction (Bao et al., 1999).

*DNA extraction, purification and quantification*

Soil microbial DNA was directly extracted from each soil sample by freeze-grinding mechanical lysis as previously described (Zhou et al., 1996). The freshly extracted DNA was purified twice using 0.5% low melting point agarose gel followed by phenol-chloroform-butanol extraction. DNA quality was assessed and final DNA concentrations were quantified with a PicoGreen methodusing a FLUO star Optima (BMG Labtech, Jena, Germany) (Ahn et al., 1996).

*DNA sequencing*

Based on the V4 hypervariable region of bacterial 16S rRNAs, the PCR primers, F515: GTGCCAGCMGCCGCGG, and R806: GGACTACHVGGGTWTCTAAT were selected and tagged (Caporaso et al., 2011; Caporaso et al., 2012). The amplicon size is 253 bp (not including the primers). The amplification mix contained 10 units of AccuPrime High Fidelity *Taq* polymerase (Invitrogen, Grand Island, NY), 2.5 µl AccuPrime PCR reaction buffer, 200 µM dNTPs (Amersham, Piscataway, NJ), and a 0.2 µM concentration of each primer in a volume of 25µl. Genomic DNA (10ng) was added to the PCR mix. Each sample was amplified under following: 30 cycles of denaturation at 95^o^C for 20s, 53^o^C for 25 s, and extension at 68^o^C for 45s, a final 10 min extension at 68 ^o^C. The PCR products were purified and collected by agarose gel electrophoresis. Denaturation was performed by 0.1M NaOH. Finally, the denatured DNA was run on a Miseq Benchtop for 2 X 150 bp paired-end sequencing (Illumina, San Diego, USA).

*Sequence data processing*

The raw sequence data were collected in Miseq sequencing machine in fastq format. The forward, reverse directions and barcodes were generated into separated files. First, the sequences were assigned to samples according to the barcodes. Paired end reads were merged into full length sequences by using FLASH program (Magoc et al., 2011). Any joined sequences with an ambiguous base were discarded. Chimera detection and removal was completed using U-Chime (Edgar et al., 2011). All sequences were clustered using UCLUST software at 97% similarity level (Edgar, 2010), and taxonomic assignment was through the Ribosomal Database Project classifier with minimal 50% confidence estimates (Wang et al., 2007). Singletons were removed for downstream analyses. All the 16S rRNA sequences were deposited in GenBank database and the accession number is SRP035449.

*Statistical analysis*

To standardize samples, a sub-sample of 20,000 sequences (nearly the fewest among the 32 samples) per soil sample was used. The number of operational taxonomic unit (OTUs) and sequences detected at different levels of classification were counted. Rarefaction curve and Chao1 indices were analyzed using Mothur software (Schloss et al., 2009). The nature and structure of the microbial community was calculated using relative abundance, Simpson’s reciprocal (1/D) and Shannon (H’) index. Detrended Correspondence Analysis was used to determine the difference of overall microbial community structure among the four different forest types analyzed here. The Multi-Response Permutation Procedure(McCune et al., 2002), Adonis (Anderson, 2001), and similarity (Anoism) (Anderson, 2001) were used to examine whether significant differences existed in the soil microbial communities among these sites. The beta-diversity was calculated using Jaccard and Bray-Curtis indices. A Mantel Test, canonical correspondence analysis (CCA) and variation partitioning analysis were used to evaluate the linkages between microbial community structure and environmental factors. All the analyses were performed by functions in the Vegan package (v.1.15-1) in R (v.2.9.1) (<http://www.r-project.org/>).

Table S1. The classified phylotypes detected at different taxonomical levels

|  | Phylum | Class | Order | Family | Genus |
| --- | --- | --- | --- | --- | --- |
| No. detected phylotpes | 36 | 90 | 153 | 275 | 1029 |
| EBF1050 | 34 | 85 | 145 | 260 | 893 |
| DBF1750 | 35 | 86 | 143 | 252 | 861 |
| CF2550 | 36 | 88 | 140 | 248 | 850 |
| SAS2750 | 35 | 86 | 142 | 241 | 825 |

Table S2. Relative abundances of detected phylum in four forest sites at different elevation

| Domain and phylum | Average^a^ (%) | | | |
| --- | --- | --- | --- | --- |
|  | EBF1050 | DBF1750 | CF2550 | SAS2750 |
| *Acidobacteria* | 18.75±2.52b | 14.23±0.69a | 13.98±1.21a | 21.34±0.75c |
| \| *Actinobacteria* \| \| --- \| | 11.80±1.48c | 9.61±0.71bc | 5.72±0.85a | 8.42±0.88b |
| \| *Armatimonadetes* \| \| --- \| | 0.14±0.01a | 0.12±0.01a | 0.10±0.01a | 0.11±0.01a |
| \| *Bacteroidetes* \| \| --- \| | 3.62±0.61a | 2.62±0.40a | 2.74±0.27a | 4.63±0.45a |
| BRC1 | 0.02±0.00a | 0.01±0.00a | 0.01±0.00a | 0.01±0.00a |
| *Chlamydiae* | 0.10±0.02a | 0.15±0.02a | 0.16±0.01a | 0.24±0.03b |
| *Chloroflexi* | 0.84±0.12a | 0.46±0.42a | 0.99±0.12a | 4.15±0.39b |
| *Cyanobacteria* | 0.06±0.01b | 0.03±0.01a | 0.04±0.01a | 0.03±0.01a |
| *Euryarchaeota* | 0.64±0.05b | 0.41±0.05a | 0.62±0.07b | 0.46±0.08a |
| *Firmicutes* | 2.59±0.30a | 2.09±0.27a | 1.87±0.13a | 3.18±1.29a |
| *Gemmatimonadetes* | 0.64±0.05b | 0.41±0.05a | 0.62±0.07b | 0.46±0.08ab |
| *Nitrospirae* | 0.11±0.02b | 0.02±0.01a | 0.09±0.02b | 0.04±0.01a |
| *Planctomycetes* | 4.50±0.31bc | 5.09±0.35c | 2.05±0.26a | 3.78±0.29b |
| *Alpha-protecobacteria* | 17.78±1.20c | 17.77±1.00c | 10.24±0.84a | 14.95±0.59b |
| *Beta-protecobacteria* | 10.41±2.42a | 15.62±2.25a | 36.41±3.96b | 10.43±1.56a |
| *Delta-proteobacteria* | 3.13±0.19b | 2.65±0.17b | 1.69±0.22a | 1.51±0.15a |
| *Gamma-proteobacteria* | 6.16±0.74a | 4.98±0.41a | 7.17±0.82a | 11.36±2.05b |
| Op11 | 0.01±0.00a | 0.01±0.00a | 0.01±0.00a | 0.01±0.00a |
| *Verrucomicrobia* | 9.19±1.61a | 17.63±2.35b | 9.31±1.11a | 8.51±1.24a |
| WS3 | 0.08±0.02a | 0.06±0.01a | 0.12±0.02b | 0.05±0.01a |
| Total Unclassified | 9.66±0.76b | 6.37±0.14a | 6.23±0.44a | 6.41±0.50a |

^a^Data represent the mean value and standard error of relative abundance detected using 8 samples in different forest sites.

Table S3. Numbers of detected OTUs at phylum level in four forest sites

| Domain and phylum | Total^a^ | Average^b^ | | | |
| --- | --- | --- | --- | --- | --- |
|  |  | EBF1050 | DBF1750 | CF2550 | SAS2750 |
| *Acidobacteria* | 12762 | 1495.25±149.5b | 1163.00±33.56a | 1135.13±64.39a | 1322.25±35.38ab |
| \| *Actinobacteria* \| \| --- \| | 7418 | 989.13±107.45c | 726.75±32.79b | 513.63±79.16a | 545.88±48.00ab |
| \| *Armatimonadetes* \| \| --- \| | 285 | 22.25±1.96b | 20.13±2.39ab | 16.75±1.76ab | 15.75±1.89a |
| \| *Bacteroidetes* \| \| --- \| | 2868 | 347.00±34.05b | 277.00±37.88ab | 247.50±17.92a | 203.88±18.13a |
| BRC1 | 64 | 2.88±0.58b | 1.63±0.46ab | 1.63±0.50ab | 0.75±0.25a |
| *Chlamydiae* | 551 | 19.75±4.58a | 27.25±2.75a | 27.38±2.30a | 41.75±5.66b |
| *Chloroflexi* | 1684 | 115.13±13.56b | 63.50±4.42a | 103.50±7.70b | 230.88±14.81c |
| *Cyanobacteria* | 58 | 6.75±1.45b | 3.38±0.50a | 4.88±0.92ab | 3.63±0.68a |
| *Euryarchaeota* | 43 | 1.25±0.45a | 1.25±0.53a | 4.63±1.16b | 3.25±0.84ab |
| *Firmicutes* | 1492 | 172.63±11.95b | 114.88±8.05a | 102.13±4.84a | 110.75±14.38a |
| *Gemmatimonadetes 675* | | 77.38±6.27b | 53.38±5.11a | 75.63±8.82b | 45.88±6.30a |
| *Nitrospirae* | 57 | 9.25±1.75b | 2.88±0.77a | 6.38±0.63b | 3.13±0.52a |
| *Planctomycetes* | 6077 | 609.38±34.13c | 634.00±18.14c | 288.00±30.81a | 375.75±27.02b |
| *Alpha-protecobacteria* | 13341 | 1418.88±79.62c | 1449.50±46.86c | 969.13±61.85a | 1225.13±35.14b |
| *Beta-protecobacteria* | 7471 | 585.38±59.07b | 746.25±79.20b | 1135.25±68.55c | 388.13±32.17a |
| *Delta-proteobacteria* | 2988 | 364.88±13.80d | 307.00±22.26c | 218.25±19.20b | 160.00±12.55a |
| *Gamma-proteobacteria* | 5348 | 441.25±10.57ab | 382.25±11.47a | 392.25±27.76a | 468.88±23.70b |
| *OP11* | 32 | 1.63±0.75b | 1.50±0.50b | 0.25±0.16a | 0.25±0.16a |
| *Verrucomicrobia* | 4890 | 519.75±64.65a | 784.00±63.52b | 540.50±25.69a | 446.38±39.19a |
| WS3 | 90 | 11.63±1.51b | 9.50±0.94ab | 14.63±2.27c | 6.00±1.07a |
| Total Unclassified | 8845 | 791.13±32.94b | 569.63±13.75a | 583.75±35.65a | 517.88±31.96a |

^a^ Data represent total numbers of detected OTUs by Illumina-sequencing across all 32 samples.

^b^ Data represent the mean value and standard error of detected OTUs using 8 samples in different forest sites.

Table S4. The OTU number of the top 10 dominant phylotypes detected at different taxonomical levels

| Different taxonomical level | EBF1050 | DBF1750 | CF2550 | SAS2750 |
| --- | --- | --- | --- | --- |
| **Class** |  |  |  |  |
| *Alphaproteobacteria* | 1478.25±41.37 | 1348±29.18 | 1183.25±53.27 | 1125.75±38.31 |
| *Betaproteobacteria* | 928.63±35.43 | 836.50±20.79 | 727.25±30.09 | 699.75±21.77 |
| *Actinobacteria* | 796.13±28.78 | 739.63±16.12 | 650.63±25.20 | 631.38±18.08 |
| *Gammaproteobacteria* | 756.13±27.73 | 682.00±16.72 | 603.00±28.31 | 574.75±19.93 |
| *Deltaproteobacteria* | 447.00±20.36 | 423.38±12.86 | 373.00±17.98 | 349.50±12.94 |
| *Spartobacteria* | 343.38±13.20 | 329.88±7.22 | 273.50±11.62 | 261.13±7.33 |
| *Acidobacteria Gp6* | 309.25±8.97 | 262.13±3.50 | 241.88±8.22 | 226.50±6.24 |
| *Acidobacteria Gp1* | 279.75±9.78 | 273.88±9.41 | 223.75±9.15 | 219.25±7.86 |
| *Sphingobacteria* | 276.63±6.43 | 236.25±7.33 | 212.00±11.83 | 188.63±5.42 |
| *Hyphomicrobiaceae* | 140.50±4.46 | 131.75±4.04 | 113.88±5.25 | 116.63±3.13 |
| **Order** |  |  |  |  |
| *Rhizobiales* | 810.88±22.68 | 743.38±14.71 | 648.63±27.40 | 641.13±23.30 |
| *Burkholderiales* | 690.75±27.55 | 614.75±16.17 | 523.63±23.77 | 509.38±17.13 |
| *Planctomycetales* | 638.13±21.16 | 581.00±19.11 | 506.63±21.87 | 461.88±13.25 |
| *Actinobacteridae* | 481.63±17.90 | 451.25±10.61 | 385.88±14.93 | 365.88±11.37 |
| *Rhodospirillales* | 359.00±9.55 | 322.50±7.09 | 287.00±16.46 | 259.75±10.97 |
| *Spartobacterias* | 338.88±13.00 | 325.63±7.54 | 270.75±11.27 | 258.13±7.28 |
| *Acidobacteria Gp6* | 309.25±8.97 | 262.13±3.50 | 241.88±8.22 | 226.50±6.24 |
| *Sphingobacteriales* | 276.63±6.43 | 236.25±7.33 | 212.00±11.83 | 188.63±5.42 |
| *Xanthomonadales* | 248.63±10.76 | 224.00±7.43 | 212.13±12.48 | 193.88±6.81 |
| *Myxococcales* | 247.75±11.37 | 225.13±8.52 | 199.75±10.40 | 196.63±8.58 |
| **Family** |  |  |  |  |
| *Planctomycetaceae* | 638.13±21.16 | 581.00±19.11 | 506.63±21.87 | 461.88±13.25 |
| *Actinomycetales* | 481.25±17.94 | 450.13±10.73 | 384.50±14.85 | 363.88±11.49 |
| *Oxalobacteraceae* | 466.13±20.28 | 408.00±11.72 | 348.13±18.18 | 337.13±11.39 |
| *Bradyrhizobiaceae* | 296.00±8.88 | 260.13±5.36 | 231.38±10.87 | 231.50±7.83 |
| *Rhodospirillaceae* | 194.13±5.58 | 167.00±6.36 | 149.50±9.96 | 130.88±6.09 |
| *Xanthomonadaceae* | 189.13±8.78 | 170.50±5.75 | 160.63±10.18 | 145.50±5.69 |
| *Chitinophagaceae* | 187.38±6.16 | 159.75±5.85 | 141.25±7.12 | 136.50±3.00 |
| *Acetobacteraceae* | 164.88±4.50 | 155.50±3.46 | 137.50±7.07 | 128.88±5.87 |
| *Solirubrobacterales* | 160.25±6.84 | 139.63±4.93 | 126.13±8.69 | 120.63±3.90 |
| *Hyphomicrobiaceae* | 140.50±4.46 | 131.75±4.04 | 113.88±5.25 | 116.63±6.41 |

Data represent the mean value and standard error of detected OTUs using 8 samples in different forest sites.

Table S5. Statistical analysis of differences in the microbial community composition and structure between different sites

| Sites | MRPP | | anosim | | adonis | |
| --- | --- | --- | --- | --- | --- | --- |
|  | δ | p | R | p | R^2^ | p |
| EBF1050-DBF1750 | 0.632 | 0.002 | 0.581 | 0.004 | 0.226 | 0.001 |
| EBF1050-CF2550 | 0.602 | 0.001 | 0.895 | 0.001 | 0.355 | 0.001 |
| EBF1050-SAS2750 | 0.612 | 0.001 | 0.919 | 0.001 | 0.383 | 0.001 |
| DBF1750-CF2550 | 0.553 | 0.001 | 0.911 | 0.001 | 0.283 | 0.001 |
| DBF1750-SAS2750 | 0.563 | 0.001 | 0.988 | 0.001 | 0.358 | 0.001 |
| CF2550-SAS2750 | 0.533 | 0.001 | 0.978 | 0.001 | 0.363 | 0.001 |

Table S6 Microbial beta-diversity of Jaccard and Bray-Curtis index along elevational distance on Shennongjia Mountain

| Sites | Jaccard beta-diversity | Bray-Curtis beta-diversity |
| --- | --- | --- |
| EBF1050-EBF1050 | 0.63 | 0.50 |
| DBF1750-DBF1750 | 0.60 | 0.46 |
| CF2550-CF2550 | 0.57 | 0.42 |
| SAS2750-SAS2750 | 0.52 | 0.43 |
| EBF1050-DBF1750 | 0.82 | 0.70 |
| EBF1050-CF2550 | 0.87 | 0.71 |
| EBF1050-SAS2750 | 0.91 | 0.84 |
| DBF1750-CF2550 | 0.78 | 0.64 |
| DBF1750-SAS2750 | 0.83 | 0.72 |
| CF2550-SAS2750 | 0.80 | 0.66 |

Fig. S1 Rarefaction curves for OTUs were calculated with sequences normalized to 20,000 for each sample using 0.03 distance OTUs.

Figure. S2 The regression relationship between soil microbial OTUs richness and plant diversity.

Figure. S3 The regression relationship between soil microbial OTUs richness and soil pH.

**References**

Ahn, S., Costa, J., Emanuel, J. (1996) PicoGreen quantitation of DNA: effective evaluation of samples pre- or post-PCR. Nucleic Acids Res., 24: 2623-2625.

Anderson, M. J. (2001) A new method for non-parametric multivariate analysis of variance. Australian Ecology, 26: 32-46.

Bao, S. D. (1999) Soil and agricultural chemistry analysis. Beijing: China Agriculture Press, 25-150.

Caporaso, J. G., Lauber, C. L., Walters, W. A., Berg-Lyons, D., Lozupone, C. A., Turnbaugh, P. J., Fierer, N., Knight, R. (2011) Global patterns of 16S rRNA diversity at a depth of millons of sequences per sample. PNAS, 108: 4516-4522.

Caporaso, J. G., Lauber, C. L., Walters, W. A., Berg-Lyons, D., Huntley, J., FIerer, N., Owens, S. M., Betley, J., Fraser, L., Bauer, M., Gormley, N., Gilbert, J. A., Smith, G., Knight, R. (2012) Ultra-high throughput microbial community analysis on the Illumina Hiseq and Miseq platforms. ISME J, 6: 1621-1624.

Edgar, R. C. (2010) Search and clustering orders of magnitude faster than BLAST. Bioinformatics, 26: 2460-2461.

Edgar, R. C., Haas, B. J., Clemente, J. C., Quince, C., Knight, R. (2011) UCHIME improves sensitivity and speed of chimera detection. Bioinformatics, 27: 2194-2200.

Ma, C., Zhu, C., Zheng, C., Wu, C., Guan, Y., Zhao, Z. (2008) High-resolution geochemistry records of climate changes since late-glacial from Dajiuhu peat in Shennongjia Mountains, Central China. Chinese Science Bulletin, 53 (Supp.1): 28-41.

Magoč T, Salzberg SL: FLASH: Fast Length Adjustment of Short Reads to Improve Genome Assemblies. Bioinformatics. 2011, 27(21): 2957-2963.

McCune B, Grace JB: Analysis of ecological communities. MJM Software Design, Gleneden Beach, OR.

Myers, R. T., Zak, D. R., White, D. C., Peacock, A. (2001) Landscape-level patterns of microbial community composition and substrate in upland forest ecosystems. Soil Sci Soc Am J, 65: 359-367.

Schloss, P. D., Westcott, S. L., Ryabin, T., Hall, J. R., Hartmann, M., Hollister, E. B., Lesniewski, R. A., Oakley, B. B., Park, D. H., Robinson, C. J., Sahl, J. W., Stres, B., THallinger, G. G., Van Horn, D. J., Weber, C. F. (2009) Introducing mothur: Open-source, platform-independent, community-supported software for describing and comparing microbial communities. Appl Environ Microbiol, 75(23): 7537-41

Wang, Q., Garrity, G. M., Tiedje, J. M., Cole, J. R. (2007) Naïve Bayesian classifier for rapid assignment of rRNA sequences into the new bacterial taxonomy. Applied and Environmental Microbiology, 73: 5261-5267.

Zhou, J. Z., Bruns, M. A., Tiedje, J. M. (1996) DNA recovery from soils of diverse composition. Appl Environ Microbiol , 62: 316-322.

Zhao, C., Chen, W., Tian, Z., Xie, Z. (2005) Altitudinal pattern of plant species diversity in Shennongjia Mountains, Central China. Journal of Integrative Plant Biology, 47(12): 1431-1449.
